# Emotion recognition in patients with mild cognitive impairment: The role of face processing and emotional intelligence

**DOI:** 10.1177/13872877251414969

**Published:** 2026-02-02

**Authors:** Rachana Mahadevan, Naomi Kristin Giesers, Thomas Liman, Karsten Witt, Andrea Hildebrandt, Mandy Roheger

**Affiliations:** 1 Ambulatory Assessment in Psychology Lab, Department of Psychology, Carl von Ossietzky Universität Oldenburg, Oldenburg, Germany; 2 University Clinic for Neurology, Department of Human Medicine, Carl von Ossietzky Universität Oldenburg, Oldenburg, Germany; 3 Center for Stroke Research Berlin, Charité – Universitätsmedizin Berlin, Berlin, Germany; 4 German Center for Neurodegenerative Diseases (DZNE), Berlin, Germany; 5 Research Center Neurosensory Science, University of Oldenburg, Oldenburg, Germany; 6 Psychological Methods and Statistics, Department of Psychology, Carl von Ossietzky Universität Oldenburg, Oldenburg, Germany

**Keywords:** Alzheimer's disease, emotional intelligence, emotion recognition, face processing, mild cognitive impairment

## Abstract

**Background:**

Emotion recognition ability is essential for social cognition, enabling humans to interpret and respond to emotion-related cues. However, so far, it is not known how underlying cognitive deficits, face processing and emotional intelligence are associated with emotion recognition, particularly in patients with mild cognitive impairment (MCI).

**Objective:**

To examine emotion recognition performance in patients with MCI and the associations between emotion recognition, face processing, emotional interference, and emotional intelligence.

**Methods:**

60 participants (patients with MCI = 30, healthy controls (HC) = 30), aged 50–86 years (M = 66.8, SD = 8.66), completed the Emotion Composite Task (ECT), Facial Composite Task (FCT), and Emotion Stroop Task. Emotional intelligence (EI) was assessed using the Trait Emotional Intelligence Questionnaire (TEIQUE).

**Results:**

Overall, patients with MCI performed worse on the ECT than healthy controls (β = −0.36, *p* = 0.207, FDR *p* = 0.311), although this difference did not reach significance. Emotion-specific analysis showed that anger recognition was particularly impaired in patients with MCI (β = −0.86, *p* < 0.001). Better face processing ability was associated with better anger recognition (β = 0.28, *p* < 0.05), and higher EI with better overall emotion recognition (β = 0.62, *p* < 0.05).

**Conclusions:**

Although overall emotion recognition performance did not significantly differ between groups, patients with MCI showed selective impairments in recognizing anger. Face processing and emotional intelligence were associated with better emotion recognition, suggesting that patients with MCI who have stronger perceptual and socio-emotional skills preserve their emotion recognition abilities more effectively.

## Introduction

Emotions determine how we respond to immediate challenges that affect our survival or matters that concern our overall well-being.^1^ Despite there being various cultural and social factors that influence emotional experiences and their expression, several studies support the notion of six universally recognized emotions, namely: anger, fear, happiness, sadness, disgust, and surprise.^2^ To understand the emotions that another person is expressing, we need to be able to process, perceive, and interpret the cues that are available to us.^3^ Important, but not exclusive, emotional signals are conveyed through nonverbal facial expression cues (for example, raising eyebrows in anger).^4^ Therefore, to interact successfully with our peers, emotion recognition, the ability to perceive and categorize emotions, and the ability to infer people's internal states are needed.

While emotion recognition impairments are well documented in dementia and particularly in Alzheimer's disease (AD),^5,6^ only a few studies have highlighted these deficits in mild cognitive impairment (MCI). According to Peterson et al.,^7,8^ MCI is a syndrome in which individuals report a cognitive complaint, have a greater cognitive decline in various domains such as attention, language, memory, executive functioning, and visuospatial construction as compared to other individuals who belong to the same age group and educational level.^9^ It is, however, important to note that patients with MCI—in contrast to patients with dementia—are still capable of independently performing their daily activities.^7^ Compared with healthy controls, patients with MCI have been shown in the literature to have impairments in emotion recognition,^10^ especially in recognizing negative emotions such as anger, fear, and sadness. In addition, the reduced ability to recognize emotions is a significant problem for the patient's caregiver and can lead to caregiver burden.^11,12^

A reasonable explanation is that patients with MCI tend to have deficits in cognitive domains that influence their performance in several tasks. These deficits overlap with the domains that play a major role in emotion recognition, such as memory, attention, language, executive functioning, and visuospatial construction,^13^ leading to deficits in recognizing emotions accurately. In addition, emotional intelligence, which includes the ability to perceive, understand, and manage emotions, has been identified as an important factor in social interactions. Emotion recognition is considered a key component in this framework. Hildebrandt et al. (2015)^14^ state that the ability to perceive and remember emotional expressions is a key aspect of emotional intelligence, suggesting that deficits in this ability may not only affect social functioning but also indicate impairments in broader cognitive abilities.

Based on the available literature, there is evidence of changes in patients with MCI, suggesting that they may have difficulty in processing different facial features,^15^ and hence raising questions about their ability to integrate these features into a coherent whole. This process is known as configural face processing and is needed for a holistic representation of the face.^16^ It is widely agreed that we process most objects based on their individual parts,^16^ especially emotion recognition which heavily relies on understanding the spatial relationships between different facial features.^17^ For example, when we describe that someone is happy, we often focus on the smile of the individual, which includes the mouth as well as other features such as the eyes and the cheeks. Hence, the deficits in configural face processing may contribute to emotion recognition difficulties in patients with MCI. However, it remains unclear whether structural changes are specifically causing deficits in emotion recognition or whether they reflect broader impairments in face processing. To understand the nature of emotion recognition deficits in MCI in association with overall face processing, it is essential to address this question at the behavioral first.

Thus, this study aims to gain a deeper understanding of the emotion recognition abilities of patients with MCI compared to healthy controls (HC). Unlike previous research, we include both an emotion recognition task and a face processing task to bridge the gap between recognition of emotional and non-emotional faces, given that the two were shown to be highly associated with younger individuals.^14^ This approach allows us to investigate whether impairments in MCI are specific to emotional expressions or reflect broader difficulties in face processing, thus addressing a critical gap in existing studies.^18^ In addition to the facial stimuli (both emotion and non-emotion related), we used stimuli comprised of words (both emotion and non-emotion related) to assess how emotional interference is associated with the ability to focus on a primary task such as reading and comprehension to gain an insight into the emotion-cognition interactions in language processing and its alterations in MCI.

The behavioral assessment battery included several tasks such as the Emotion Composite Task (ECT), Facial Composite Task (FCT), and Emotion Stroop Task to measure emotion recognition, facial processing, and emotional interference (Emotional Stroop Effect, ESE), respectively. Hence, we expect that (1) patients with MCI will show reduced emotion recognition performance compared to HC, indicating that patients with MCI have a reduced ability to attend to emotional cues. Furthermore, we expect that (2) in patients with MCI, the association of face processing to emotion recognition will be stronger than in HCs. Finally, we expect (3) that higher emotional interference to emotional words is associated with lower emotion recognition in patients with MCI, but less in HCs. The association of additional predictors such as age, gender, emotional intelligence, and neurocognitive test scores in domains such as memory, attention, executive function, and language with emotion recognition were investigated across groups.

## Methods

The current study investigates two main groups of individuals: HC and patients with MCI. The study was pre-registered (https://osf.io/yn3gp). Reporting follows the TREND Statement Checklist in Supplemental Table 1.^19^

### Participants

The study was carried out in the Department of Psychology at the University of Oldenburg, Germany, from October 2023 until December 2024. Patients were recruited at the Evangelisches Krankenhaus Oldenburg. A total of 60 participants belonging to the age range of 50 to 86 years (*M* = 67, *SD* = 8.7) were enrolled in the study, among them Healthy Controls (n = 30; age: *M* = 63, *SD* = 7.6; 17 females, 13 males) and patients with MCI (n = 30; age: *M* = 70 *SD* = 8.2; 14 females, 16 males). Initially, we intended to recruit both patients with amnestic MCI (aMCI; single domain aMCI, which indicates a memory decline, and multiple domain aMCI, in which decline occurs in other cognitive domains in addition to memory^8^) as well as patients with non-amnestic MCI (naMCI; single domain naMCI, indicating a decline in one cognitive domain other than memory, or multiple domain naMCI, in which decline occurs in more than one cognitive domain other than memory^8^). Unfortunately, we were unable to sample enough participants with naMCI in a reasonable timeframe, thus the present analysis will not focus on this distinction. In total, among the 30 patients with MCI included in the study, 25 were classified as amnestic MCI (10 single-domain, 15 multi-domain) and 5 as non-amnestic MCI (4 single-domain, 1 multi-domain). Due to the small number of naMCI participants, all MCI subtypes were analyzed together to ensure sufficiently larger group size. The DSM criteria for the diagnosis of MCI includes (a) memory complaints that are reported subjectively either by the patients or their family members (b) the ability to perform normal activities of daily living without interruption as documented by the Instrumental Activities of Daily Living (IADL)^20^ scale (c) a slight decline in general cognitive functions that can be objectively measured through the Montreal Cognitive Assessment (MoCA)^21^ with cut off scores ranging from 18–25 (d) and the absence of dementia.

**Figure 1.**
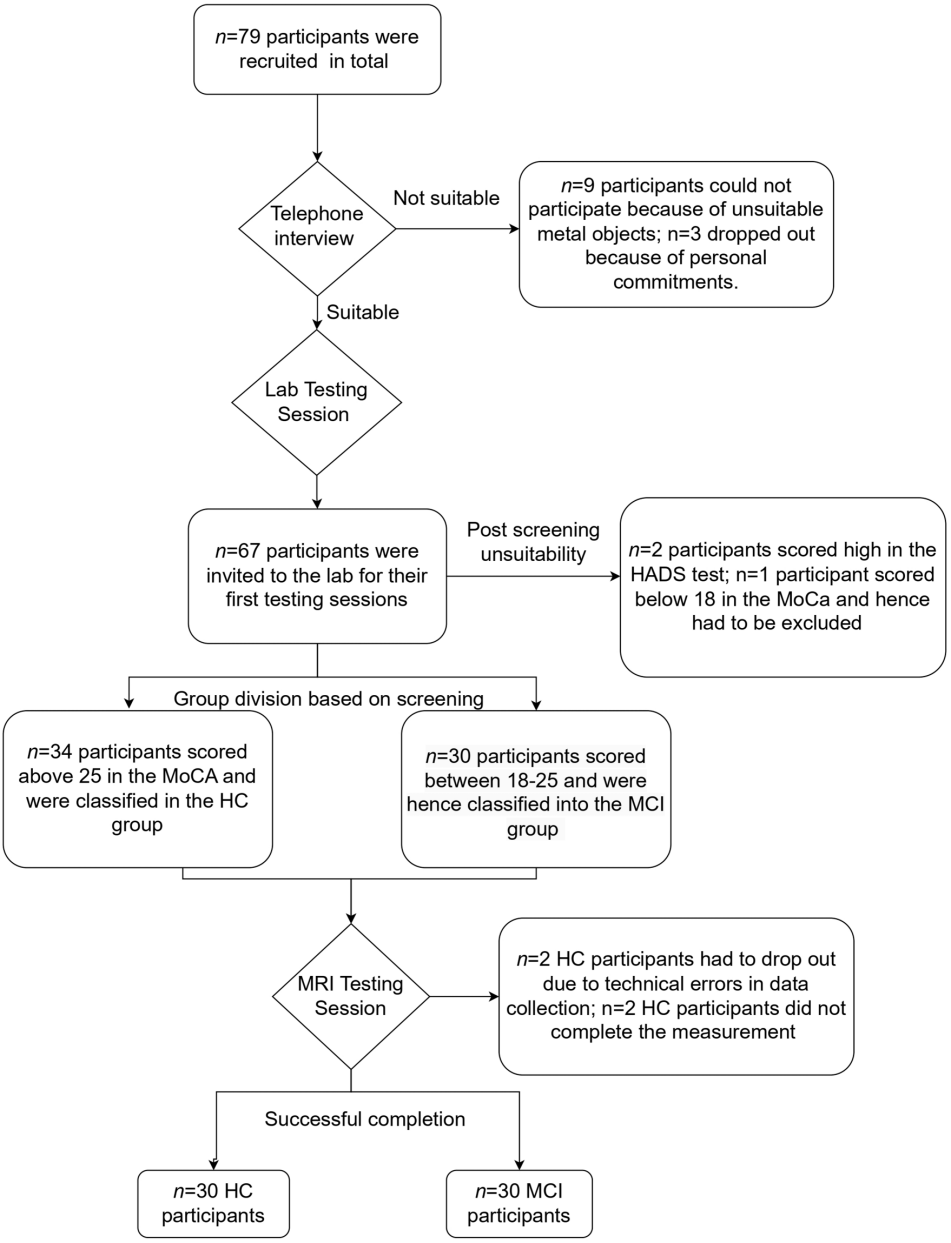
Participant recruitment process from the screening stage through the testing stages.

The inclusion criteria of the study ensured that the participants were 50 years or above in age, fluent in German, did not have any known psychiatric or neurological disorders, and had an absence of substance dependency in the last 6 months. Exclusion criteria were based on scores obtained in the Instrumental Activities of Daily Living (IADL)^20^ questionnaire (participants had to score 6 points or above to indicate that they could function independently) and the cut-off scores obtained from the Hospital Anxiety and Depression Scale (HADS)^22^ for HCs was limited to 8 points, and for patients with MCI was limited to 11 points.^23^ This was to rule out the possibilities of existing anxiety and depression that could alter the cognitive task performances. Further, participants were excluded if they had an existing dementia diagnosis and if they had implanted devices or metal objects that were not suitable for the magnetic resonance imaging machine. Although this study also collected MRI data, this paper focuses on the behavioral task results. The MRI results that explore the structural and functional brain characteristics of emotion recognition in MCI are reported elsewhere. A detailed summary of the flow of participation is shown in Figure 1.

### Procedure

During the initial recruitment phases, we conducted telephone interviews with potential participants to check their eligibility. Once the criteria were met, we invited the participants to the lab for the first testing session. In this session, a written informed consent form was presented, and the participants signed it once they were fully informed about the procedure. This session included neuropsychological assessments and an emotional intelligence questionnaire; the details of these tests are described below in the materials section. Next, the Facial Composite Task (FCT), the Emotion Composite Task (ECT), and the Emotion Stroop Task were conducted, all of which were administered via a computer. In the end the participants were invited to the MRI session.

### Materials

*Neuropsychological assessment.* As a part of the screening procedure, we administered the IADL^20^ to assess participants’ ability to perform daily activities independently, followed by the HADS^22^ to further ensure that they did not suffer of anxiety or depression, and finally, MoCA^21^ to distinguish between HCs (cut off includes scores 26 and above) and patients with MCI (with cut off scores ranging from 18 to 25). In addition, we administered the Consortium to Establish a Registry for Alzheimer's Disease Neuropsychology Battery (CERAD-NAB),^24^ a neuropsychological battery used in German-speaking countries, and adjusted for age, gender, and education in an elderly population. Detailed descriptions of these assessments can be found in Supplemental Table 2.

*Emotional intelligence.* To measure the Emotional Intelligence (EI), we administered the Trait Emotional Intelligence Questionnaire – Short Form (TEIQUE-SF),^25^ which consisted of 30 questions and measured four facets of EI: Well-Being, Self-Control, Emotionality, and Sociability. The participants responded via a Likert scale with 7 options ranging from strongly disagree to strongly agree. The responses can be summarized by forward and reverse scoring, and this was conducted via the scoring engine on TEIQUE's website. The interpretation of the scores are explained in Supplemental Table 3.

**Figure 2.**
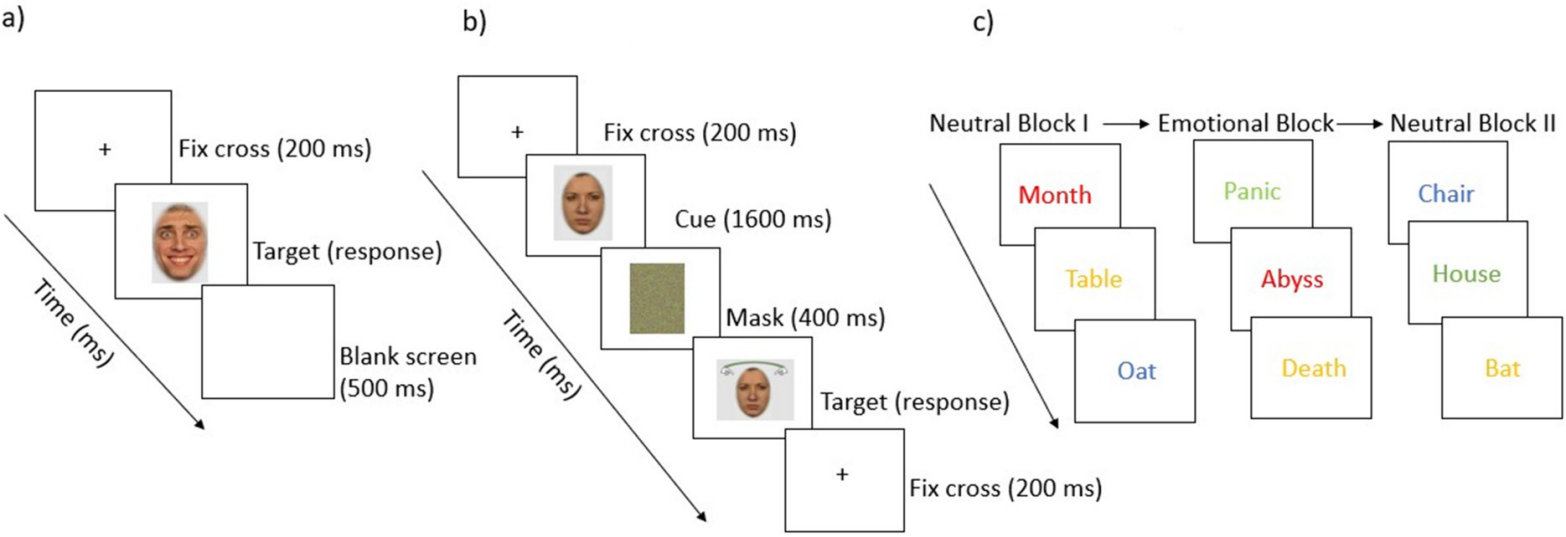
Overview of the experimental tasks: a) Emotion Composite Task, b) Facial Composite Task, and c) Emotional Stroop Task.

*Facial composite task.* To measure facial processing ability, we administered the FCT task similar to Petrakova et al. (2018).^26^ The test was programmed in the Presentation^®^ software (version 23.0, Neurobehavioral Systems, Inc., Berkeley, CA, www.neurobs.com). We presented 40 trials with 6 practice rounds. Participants were asked to fixate the screen with their gaze, where a fixation cross was displayed for 200 ms, followed by a first composite face for 1600 ms (composite faces were created by aligning the top half of one face with the bottom half of another and presented upright"^27^). Participants were requested to pay attention to this cue. A green mask then appeared on the screen for a brief period of 400 ms. This was followed by a second composite face (target) that was included with two hands appearing either at the top or bottom of the face, and this target remained on the screen until the participant responded (see also Figure 2).

The task was specifically designed to ensure that participants attended to both halves of the face and were able to switch their attention to the half where the hands were displayed. Between trials, a black screen was displayed for 500 ms. Participants could respond using marked keys to indicate whether they thought that the displayed half of the face was the same or different from the half of the face that was shown first.

*Emotion composite task.* To measure the emotion recognition abilities, we obtained the Emotion composite task from Wilhelm et al. (2014).^28^ This task was based on the Composite face paradigm proposed by Calder et al. (2000)^29^ that aimed to investigate the perceptual mechanisms that underlie facial expression processing and the role of configural information in expression perception. They created the stimuli by aligning the upper and lower faces of the same person; however, each half displayed a different emotion. According to previous research by Calder et al. (2000), emotions such as fear, sadness, and anger are primarily conveyed through features in the upper half of the face (e.g., eyes and brows), whereas happiness, surprise, and disgust are more accurately identified from features in the lower half of the face (e.g., mouth and cheeks).^28^ This evidence formed the basis for the design of the current task, in which the upper halves expressing negative emotions were combined with the lower halves expressing positive emotions to systematically examine the configural processing of emotional expressions. Accordingly, the top halves expressing anger, fear, and sadness were combined with the bottom halves expressing happiness, surprise, and disgust, resulting in nine different composite stimuli. The task was programmed in PsychoPy,^30^ and the entire task consisted of 144 trials, i.e., 24 trials for each emotion. The task begins with a practice round including 6 trials and the participants had to focus on the screen so that they could pay attention to the cues that appeared on the screen, for example when the cue-word ‘UPPER’ appeared they had to select the correct emotion that the top half of the face was displaying and when the cue-word ‘LOWER’ appeared, they had to select the correct emotion that the lower half of the face was displaying. Participants could respond with a simple mouse click by selecting one of the six labeled options that appeared below the stimuli in a horizontal row, namely anger, sadness, fear, happiness, disgust, and surprise. After response, the stimuli disappeared, and the screen remained blank for 500 ms before the fixation cross for the next trial appeared on the screen (see Figure 2).

*Emotion Stroop Task.* To measure emotion-induced interference, we included the Emotional stroop task, which was designed following the guidelines by Ben Heim (2016).^31^ This task comprises three blocks consisting of either neutral or emotional words written in different colors, and it was presented in Presentation^®^ software (Version 23.0, Neurobehavioral Systems, Inc., Berkeley, CA, www.neurobs.com).

The task started with the training round to let participants familiarize themselves with the key mapping. The training block consisted of 20 neutral words and was followed by the main task. First, participants were presented with 20 neutral words that were presented in different color inks, and these words are known to induce no emotional arousal (e.g., tree, hat). Following the first neutral block, 20 emotional words were presented in different color inks (e.g., abyss, crisis). Finally, a second block of 20 neutral words was presented. The words appeared on a neutral background, and participant had to respond via keys associated with the color of the words, namely, red, blue, green, or yellow. Between each block, a break of 3000 ms, and between each trial of the blocks, a break of 600 ms was incorporated. Once the stimuli were displayed, the reaction time was measured, i.e., the amount of time the participant took to identify the color and respond using the keys (see also Figure 2). Participants who were color blind (n = 2 participants in our study did not take part due to color blindness) did not have to take part in this task, and their rows were marked as missing values in the dataset. This task intended to measure the Emotional Stroop Effect (ESE), which refers to “the phenomenon that the emotional information of stimuli will delay the reaction of participants when they are asked to respond to the non-emotional information in a task”.^32,33^ That is, in simpler terms, the ESE is the result of the longer reaction time to name the ink colors of emotional words than to name the ink colors of neutral words and is calculated by subtracting the reaction times of the emotional block from the first neutral block.

### Statistical analysis

Statistical analysis was conducted using R Software for Statistical Computing (v4.1.2; R Core Team 2021). Descriptive summary statistics were calculated for continuous variables such as age, education, and test scores (CERAD, MoCA, EI, and TEIQUE scores), and independent *t*-tests were used to compare group means (HC versus MCI) for these variables (Table 1). Frequency distributions for dichotomous variables such as group and gender are included in Supplemental Tables 4 and 5.

**Table 1.** Descriptive statistics of demographic and cognitive variables across groups.

Variable	All participants (*n* = 60) *M*	*SD*	MCI (*n* = 30)		HC (*n* = 30)		HC versus MCI	
			*M*	*SD*	*M*	*SD*	*t*-value	*p*-value
Age	66.80	8.66	70.50	8.09	63.10	7.55	−3.644	**<0**.**001**
Education	15.60	2.57	15.10	2.85	16.20	2.11	1.727	0.090
MoCA	25.40	2.71	23.10	1.55	27.70	1.25	12.572	**<0**.**001**
EI	5.51	0.70	5.27	0.74	5.74	0.58	5.740	**<0**.**05**
ECT	61.40	14.30	64.8	13.70	58.10	14.20	1.838	0.071
FCT	25.60	5.25	23.60	5.36	27.80	4.19	3.221	**<0**.**05**
Immediate Memory	−0.22	1.38	−1.04	1.09	0.59	1.15	5.564	**<0**.**001**
Delayed Memory	−0.58	0.79	−0.76	0.85	−0.41	0.67	1.787	0.080
Word Recognition	−0.34	1.07	−0.56	1.17	−0.11	0.91	1.664	0.101
Visuospatial recall	0.23	1.11	−0.16	1.30	0.62	0.67	2.931	**<0**.**05**
Visuospatial Construction	−0.01	1.06	−0.31	1.23	0.29	0.75	2.259	**<0**.**05**
Verbal Fluency	−0.11	0.98	−0.15	1.00	−0.08	0.96	0.269	0.800
Boston Naming Test	0.35	0.80	0.40	0.86	0.30	0.74	−0.504	0.616
Phonematic Fluency	0.26	0.98	0.30	1.00	0.22	0.96	−0.302	0.763
TMT A	0.77	0.98	0.70	0.93	0.84	1.00	0.541	0.590
TMT B	0.64	0.99	0.75	0.88	0.54	1.07	−0.808	0.422

*M*: mean; *SD*: standard deviation; MoCA: Montreal Cognitive Assessment; EI: Emotional intelligence; ECT: Emotional composite task; FCT: Facial composite task; TMT: Trail Making Test. The mean and standard deviation of the z-scores of the tests (intended to measure attention (TMTA), memory (Immediate memory, delayed memory, word recognition, visuospatial recall), executive functioning (TMTB), language (verbal fluency, Boston naming test and phonematic fluency), and visuospatial construction are displayed and the p values represent the group comparisons between MCI (mild cognitive impairment) and HC (Healthy controls). Statistically significant results are shown in bold (*p* < 0.001 and *p* < 0.05).

For hypotheses testing, we estimated multiple one-level general linear regression and multilevel models and performed our analysis in a stepwise approach. The one-level linear regression models were used to examine the overall group differences, while the multilevel models were used to account for within-subject differences, such as recognizing specific emotions. For hypothesis 1, we tested whether patients with MCI exhibit reduced emotion recognition performance compared to the HCs. To examine group differences (HC versus MCI), we estimated a categorical regression model with the groups as the main categorical predictor and ECT scores as a continuous outcome variable. To test group differences in recognizing different emotion categories, we estimated a multilevel model that included an interaction between emotion categories and the group membership. Dummy coding was applied with happiness as a reference (coded zero on all coding variables). For the binary grouping variable, the HC group was coded with zero and thus served as a reference category, whereas the patients with MCI were coded with 1. For hypothesis 2, we postulated that the association of face processing with emotion recognition will be stronger in HCs than in patients with MCI. To test this hypothesis, we further estimated a multilevel model that included FCT scores as a predictor, along with the interaction between each FCT and the groups, and FCT with emotion category coding variables. For hypothesis 3, we postulated that higher emotional interference to emotional words will be associated with lower emotion recognition in patients with MCI than in HCs. To test this, we estimated a multilevel model that included the ESE interference effects as a predictor, along with the interaction between each ESE and the groups, and ESE with emotions. All analyses included age as a covariate to control for group differences in age. To control for multiple comparisons, all *p*-values were adjusted using the False Discovery Rate (FDR) correction method.

As an exploratory analysis, we further estimated a series of one-level general linear regression models to examine the associations between EI, cognitive test *z*-scores (memory, attention, executive functioning, visuospatial construction, and language), and gender-stimuli interactions with emotion recognition as measured by ECT performance. We tested key assumptions for the above models, including normality of the residuals (e.g., Shapiro-Wilk test for normality), homoscedasticity (e.g., using residual plots), and multicollinearity (e.g., variance inflation factors) among predictors to ensure that the requirements are fulfilled for the linear models to be estimated. Missing values were accounted for in the analysis through listwise detection and excluded.

## Results

### Descriptive statistics

The demographic information and cognitive scores of the entire sample, including distinctive group characteristics, are summarized in Table 1. Patients with MCI were significantly older and had a lower MoCA score than HCs. Additionally, the HCs outperformed the patients with MCI in a wide range of cognitive domains tested.

### Analysis

*Group differences in emotion recognition.* Patients with MCI scored lower on the Emotion Composite Task (ECT) compared to healthy controls (β = −0.36, p = 0.207, FDR p = 0.311), indicating a small but non-significant group difference after controlling for age (Table 2). Thus, the available evidence does not support a statistically significant overall difference in emotion recognition performance between patients with MCI and healthy controls.

**Table 2.** Group differences in ECT performance.

Parameter	β	CI	*p*	FDR *p*
(Intercept)	0.18	[−0.2, 0.56]	**<0**.**001**	**<0**.**001**
Group	0.36	[−0.92, 0.2]	0.207	0.311
Age	-0.12	[−0.41, 0.16]	0.384	0.384

Residual standard error: 14.01 on 57 degrees of freedom, Multiple R-squared: 0.06761, Adjusted R-squared: 0.0349.

F-statistic: 2.067 on 2 and 57 DF, p-value: 0.136.

β displays the standardized β values, FDR is the false discovery rate, Confidence Interval (CI) = 95%, and statistically significant results are shown in bold (p < 0.001 and p < 0.05). The group variable compares the emotion recognition abilities of the patients with mild cognitive impairment (MCI) and healthy controls (HC).

**Figure 3.**
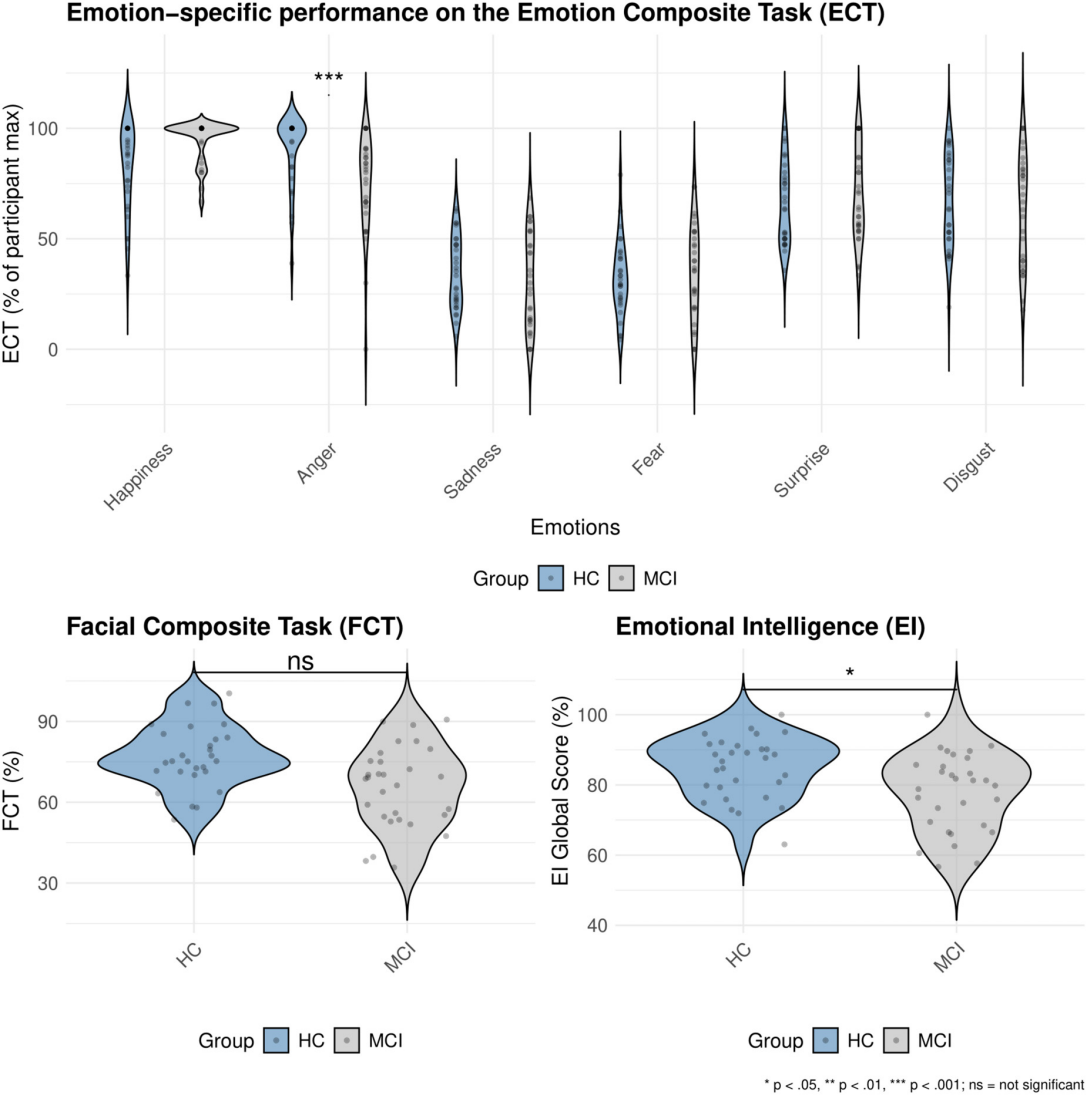
A multi-panel violin plot showing a) Emotion-specific performance on the emotion composite task (ECT) across groups (HC and MCI), b) Facial composite task (FCT) score distribution across groups (HC and MCI), c) Emotional intelligence (ei) global score distribution across groups (HC and MCI).

*Do groups differ in recognizing specific emotions?* The results of the multilevel model including interaction between the grouping variable and emotion categories predicting the ECT scores are shown in Table 3. Independently of the group, the recognition accuracy for anger was not significantly different from happiness (β = 0.25, *p* = 0.112, FDR *p* = 0.173). In contrast, recognition accuracy was lower for sadness (β = −1.59, *p* < 0.001), fear (β = −1.69, *p* < 0.001), surprise (β = −0.56, *p* < 0.001) and disgust (β = −0.58, *p* < 0.001), indicating that these emotions were significantly more difficult to recognize compared to happiness, with sadness and fear showing the largest negative effects. On the group level, we found that the patients with MCI had significantly lower accuracy in anger recognition compared to HC (β = −0.86, *p* < 0.001), indicated by a strong negative effect. The large effect size highlighted that patients with MCI had most difficulties in recognizing anger. For disgust, the negative coefficient (β = −0.44) indicated lower recognition accuracy in patients with MCI, however, this effect however did not reach statistical significance (*p* = 0.051, FDR *p* = 0.094), suggesting no clear evidence for a group difference. For emotions like sadness (β = −0.35, *p* = 0.120, FDR *p* = 0.173), fear (β = −0.12, *p* = 0.604, FDR *p* = 0.604), and surprise (β = −0.25, *p* = 0.261, FDR *p* = 0.339), the coefficients showed weak effects that did not reach statistical significance. Figure 3a displays violin plots depicting the distribution of the ECT scores across groups for different emotions.

**Table 3.** Emotion-specific performance across groups.

Parameter	β	CI	*p*	FDR *p*
(Intercept)	0.78	[0.51, 1.04]	**<0**.**001**	**<0**.**001**
Anger	0.25	[−0.06, 0.56]	0.112	0.173
Sadness	−1.59	[−1.89, −1.28]	**<0**.**001**	**<0**.**001**
Fear	−1.69	[−2.00, −1.39]	**<0**.**001**	**<0**.**001**
Surprise	−0.56	[−0.87, −0.25]	**<0**.**001**	**<0**.**001**
Disgust	−0.58	[−0.89, −0.27]	**<0**.**001**	**<0**.**001**
Group	0.17	[−0.21, 0.55]	0.377	0.413
Age	−0.06	[−0.18, 0.07]	0.381	0.413
Anger × Group	−0.86	[−1.30, −0.42]	**<0**.**001**	**<0**.**001**
Sadness × Group	−0.35	[−0.78, 0.09]	0.120	0.173
Fear × Group	−0.12	[−0.55, 0.32]	0.604	0.604
Surprise × Group	−0.25	[−0.69, 0.19]	0.261	0.339
Disgust × Group	−0.44	[−0.87, 0.00]	0.051	0.094

Random Effects: σ2 = 10.00, τ00 ID = 3.78, ICC = 0.27, N ID = 60, Observations=360, Marginal R2 / Conditional R2 = 0.498 / 0.635.

β displays the standardized values, FDR is the false discovery rate, Confidence Interval (CI) = 95%, and statistically significant results are shown in bold (*p* < 0.001 and *p* < 0.05). Emotions (anger, sadness, fear, surprise, and disgust) represent the individual scores on the Emotion composite task. The group variable compares the emotion recognition abilities of the patients with mild cognitive impairment (MCI) and healthy controls (HC). The interaction between each emotion and the groups was used to assess whether the recognition of individual emotions differed.

*Is face processing predicting emotion recognition across groups?* The results of the multilevel model including an interaction between the grouping variable and the FCT scores predicting the ECT scores showed that across all participants, the FCT was not a significant predictor of overall emotion recognition (β = −0.02, *p* = 0.899, FDR *p* = 0.899)**,** however, the interaction of FCT and emotions revealed a significant positive effect of the FCT on anger recognition (β = 0.28, *p* < 0.05, FDR *p* < 0.05), implicating that better face processing skills were associated with better performance in anger recognition. For emotions such as sadness (β = 0.12, *p* = 0.314, FDR *p* = 0.408), fear (β = 0.11, *p* = 0.327, FDR *p* = 0.408), disgust (β = 0.17, *p* = 0.146, FDR *p* = 0.307), and surprise (β = −0.03, *p* = 0.799, FDR *p* = 0.856), the effects were small and not significant, hence indicating that facial processing was not significantly associated with the recognition of these emotions. A summary of the results are presented in Table 4. However, contrary to our second hypothesis, the interaction between FCT and the group displayed that the contribution of face processing to emotion recognition was not significantly stronger in the patients with MCI (β = 0.15, *p* = 0.246, FDR *p* = 0.408). Figure 3(b) displays violin plots depicting the distribution of the FCT scores across groups (also see Supplemental Figure 1 displays the scatterplots for this analysis).

**Table 4.** FCT and group interaction.

Parameter	β	CI	*p*	FDR *p*
(Intercept)	1.00	[0.77, 1.23]	**<0**.**001**	**<0**.**001**
FCT	−0.02	[−0.28, 0.25]	0.899	0.899
Group	−0.17	[−0.41, 0.07]	0.164	0.307
Anger	−0.22	[−0.44, 0.01]	**<0**.**05**	**<0**.**05**
Sadness	−1.78	[−2.00, −1.55]	**<0**.**001**	**<0**.**001**
Fear	−1.79	[−2.02, −1.56]	**<0**.**001**	**<0**.**001**
Surprise	−0.71	[−0.93, −0.48]	0.326	0.408
Disgust	−0.82	[−1.04, −0.59]	**<0**.**05**	**<0**.**05**
Age	0.05	[−0.09, 0.2]	0.468	0.540
FCT × Group	0.15	[−0.01, 0.39]	0.246	0.408
FCT × Anger	0.28	[0.05, 0.50]	**<0**.**05**	**<0**.**05**
FCT × Sadness	0.12	[−0.11, 0.34]	0.314	0.408
FCT × Fear	0.11	[−0.11, 0.34]	0.327	0.408
FCT × Surprise	0.03	[−0.26, 0.20]	0.799	0.856
FCT × Disgust	0.17	[−0.06, 0.39]	0.146	0.307

σ^2^ = 10.25, τ00 ID = 2.89, ICC = 0.22, N ID = 58*, Observations = 348, Marginal R2 / Conditional R2 = 0.522 / 0.627.

β displays the standardized β values, FDR is the false discovery rate, Confidence Interval (CI) = 95%, and statistically significant results are shown in bold (p < 0.001 and p < 0.05). FCT represents the scores of the Facial composite task and Emotions (anger, sadness, fear, surprise and disgust) represent the individual scores on the Emotion composite task. The group variable compares the emotion recognition abilities of the patients with mild cognitive impairment (MCI) and healthy controls (HC). The interaction between each FCT and the groups was used to assess the contribution of FCT across groups in recognizing emotions. The interaction between each emotion and the groups was used to assess whether the recognition of individual emotions differed and the level to which FCT contributes to it. *n = 2 participants did not complete the FCT task due to technical errors and their missing values were accounted for in R.

*Does emotional interference predict emotion recognition across groups?* The results of the multilevel model, including an interaction between the grouping variable and the ESE reaction times predicting the ECT scores, showed that the ESE across all participants was a significant predictor of overall emotion recognition (β=-0.33, *p* < 0.05), suggesting that high interference was associated with lower emotion recognition. The model also included an interaction between ESE and individual emotions to investigate if interference affected the recognition of specific emotions. ESE was not significantly associated with most of the emotions, such as anger (β = 0.20, *p* = 0.084, FDR *p* = 138), sadness (β = 0.22, *p* = 0.057, FDR *p* = 0.106), fear (β = 0.14, *p* = 0.236, FDR *p* = 0.295), or surprise (β = 0.15, *p* = 0.201, FDR *p* = 0.275).Whereas the significant interaction between disgust and ESE (β = 0.32, *p* < 0.05, FDR *p* < 0.05) suggested that while overall ESE still negatively impacted the recognition of disgust, it was weaker compared to other emotions. The interaction between ESE and the group was not significant (β = 0.20, *p* = 0.092, FDR *p* *=* 0.138, indicating that groups showed comparable performance despite the negative impact of overall emotional interference. A summary of the results are presented in Table 5. This contradicts our hypothesis that patients with MCI would experience stronger emotional interference that would impair their emotion recognition. Instead, interference contributed similarly to the performance of both groups. Supplemental Figure 2 displays the scatterplots for this analysis.

**Table 5.** ESE and ECT performance.

Parameter	β	CI	*p*	FDR *p*
(Intercept)	0.93	[0.70, 1.15]	**<0**.**001**	**<0**.**001**
ESE	−0.33	[−0.55, −0.10]	**<0**.**05**	**<0**.**05**
Anger	−0.18	[−0.40, 0.05]	0.490	0.525
Sadness	−1.75	[−1.98, −1.52]	**<0**.**001**	**<0**.**001**
Fear	−1.74	[−1.97, −1.52]	**<0**.**001**	**<0**.**001**
Surprise	−0.70	[−0.92, −0.47]	**<0**.**001**	**<0**.**001**
Disgust	−0.79	[−1.02, −0.56]	**<0**.**001**	**<0**.**001**
Group	−0.14	[−0.40, 0.12]	0.720	0.720
Age	−0.07	[−0.2, 0.06]	0.296	0.342
ESE×Anger	0.20	[−0.03, 0.43]	0.084	0.138
ESE×Sadness	0.22	[−0.01, 0.45]	0.057	0.106
ESE×Fear	0.14	[−0.09, 0.36]	0.236	0.295
ESE×Surprise	0.15	[−0.08, 0.37]	0.201	0.275
ESE×Disgust	0.32	[0.09, 0.55]	**<0**.**05**	**<0**.**05**
ESE×Group	0.20	[−0.03, 0.43]	0.092	0.138

σ^2^ = 10.54, τ_00_(ID) = 3.74, ICC = 0.26, ID = 58, Observations = 348, Marginal R^2^ / Conditional R^2^ = 0.492 / 0.625.

β displays the standardized β values, FDR is the false discovery rate, Confidence Interval (CI) = 95%, and statistically significant results are shown in bold (p < 0.001 and p < 0.05). ESE represents the scores of the Emotional Stroop Effect and Emotions (anger, sadness, fear, surprise, and disgust) represent the individual scores on the Emotion composite task. The group variable compares the emotion recognition abilities of the patients with mild cognitive impairment (MCI) and healthy controls (HC). The interaction between each ESE and the groups was used to assess the contribution of ESE across groups in recognizing emotions. The interaction between each emotion and the groups was used to assess whether the recognition of individual emotions differed and the level to which ESE contributes to it.*n = 2 participants did not complete the ESE task due to color blindness, and their missing values were accounted for in R.

*Do cognitive and demographic factors predict emotion recognition across groups?* A series of general linear models examined the association of cognitive and demographic factors with emotion recognition. The models included neurocognitive test z-scores (for language, memory, attention, executive functioning, and visuospatial construction) and demographic factors (age, gender, and education levels) as predictors and their interaction with groups, with the ECT as the outcome. The analysis revealed that none of the variables predict emotion recognition (all p > 0.05). On the group level, the interaction effects revealed no differences between the performance of patients with MCI and HCs among age, education, gender, or cognitive performances (see Supplemental Tables 9–15). Additionally, an interaction between the participant's gender and the stimuli's gender revealed that the gender of the stimuli was not significantly associated with the participant's task performance.

*Does emotional intelligence predict emotion recognition across groups?* A general linear model examined the association between emotional intelligence and emotion recognition. The model included the global EI scores which is an average total of the subscales such as emotionality, well-being, self-control, and sociability as predictors and an interaction with the groups (see Supplemental Figures 3–9), with ECT as the outcome. As shown in Table 6, the global EI was not significantly associated with emotion recognition across all participants (β = −0.25, *p* = 0.254, FDR *p* = 0.318). However, the interaction between EI and the groups was significant (β = 0.62, *p* < 0.05, FDR *p* < 0.05), hence suggesting that the association of EI with emotion recognition differed between groups. More specifically, EI was associated with more performance in HCs than in patients with MCI. Figure 3(c) displays violin plots depicting the distribution of the EI scores across groups. Supplemental Figure 3 displays the scatterplots for this analysis (Additional scatterplots for the subtypes of EI and specific emotions are displayed in Supplemental Figures 4–9).

**Table 6.** Association of the ei and the ECT performance.

Parameter	β	CI	*p*	FDR *p*
(Intercept)	0.27	[−0.12, 0.66]	**<0**.**001**	**<0**.**001**
EI	0.25	[−0.68, 0.18]	0.254	0.318
Group	0.34	[−0.90, 0.22]	**<0**.**05**	**<0**.**05**
Age	0.10	[−0.37, 0.18]	0.494	0.494
EI × Group	0.62	[0.08, 1.16]	**<0**.**05**	**<0**.**05**

Residual standard error: 13.38 on 48 degrees of freedom, Multiple R-squared: 0.2839, Adjusted R-squared: 0.1198, F-statistic: 1.73 on 11 and 48 DF, p-value: 0.09501.

β displays the standardized values, FDR is the false discovery rate, Confidence Interval (CI) = 95%, and statistically significant results are shown in bold (p < 0.001 and p < 0.05). EI global scores represent overall Emotional Intelligence, while well-being, self-control, emotionality, and sociability are its subdomains. The group variable compares the emotion recognition abilities of the patients with mild cognitive impairment (MCI) and healthy controls (HC). The interaction between emotional intelligence and the groups was used to assess whether the relationship between emotional intelligence and emotion recognition differed.

## Discussion

The aim of this study was to examine deficits in emotion recognition abilities of patients with MCI compared to the HCs and to determine whether these deficits could be predicted by several factors, including face processing abilities, emotional intelligence, emotional interference, cognitive abilities, and demographic factors. This study adds new insights into emotion recognition in MCI and links it to underlying cognitive mechanisms such as face processing and emotional intelligence.

### Emotion recognition deficits in MCI

Consistent with the literature, our results indicate that patients with MCI have lower emotion recognition than HCs. While the overall difference in performance did not reach statistical significance, emotion-specific analysis revealed that patients with MCI had significantly lower accuracy in recognizing anger and reduced accuracy in recognizing disgust. Previous studies have shown that emotion recognition deteriorates as subjective cognitive decline progresses to MCI and AD.^34^ Interestingly, although most studies have reported that patients have difficulties with both positive and negative emotions, most of the impairments lean toward negative emotion recognition.^10,34,35^ One explanation for the greater impairment in negative emotion recognition compared to positive emotion recognition could be due to the asymmetrical representation of negative and positive emotions in tests, where there are four negatives (sadness, fear, anger, and disgust) and only two positives (happiness and surprise).^34^ A second explanation could be that the recognition of negative emotions require more fine-grained discrimination of visual cues, for example, furrowed brows for anger and widened eyes for fear, whereas positive emotions such as happiness are often easier to recognize.^34^ A third explanation could be the structural and functional changes that are often associated with neurodegenerative disorders, for example, reduction in the volume of the amygdala and prefrontal cortex, which play a key role in emotional processing.^36^ Research has shown that this impairment is greater for negative emotions,^37^ which is consistent with the understanding that one of the primary functions of these regions involves threat detection including the regulation of fight or flight responses to stimuli encountered daily life.^38–40^ Given that the recognition of negative emotions is essential to help us identify threats and avoid misinterpretations, deficits in this area could lead to an increase in intrapersonal conflict and potentially contribute to caregiver burden.^11^

### The association of face processing and emotion recognition

One of the findings of our study was that face processing was not significantly associated with overall emotion recognition. However, the interaction between face processing and emotion showed that better face processing skills were associated with better anger recognition in both groups. This suggests that the difficulties in recognizing specific emotions may be due to configural face processing, where attention is paid to specific facial regions regardless of cognitive status to recognize the holistic faces. For example, the mouth is more relevant for happiness and the eyes are more relevant for recognizing sadness and anger.^41^ Building on this, ECT, which was designed to examine the recognition of emotions from different halves of the face further supports this notion. That is, the finding that patients with MCI showed greater difficulty in recognizing anger presented in the upper half of the face suggests that impairments in face processing particularly the inability to pay attention to all the facial features may hinder their ability to detect subtle emotional cues, that would lead to challenges in their daily life.

### The association of cognitive functions and emotion recognition

Another finding was that most of the neurocognitive domains (language, verbal memory, executive function, attention, and visuospatial construction) were not significantly associated with emotion recognition after FDR correction. These findings suggest that general cognitive abilities do not play a major role in explaining variability in emotion recognition, at least not within this sample.

The association between emotion recognition and verbal memory tests was not significant, and this was consistent with previous research suggesting that verbal memory does not necessarily impair emotion recognition.^42^ Even though the association between language and emotion recognition was not significant in our study, prior research has shown that language can support labeling emotions^41^; however, interpreting facial emotions may be highly non-verbal, and hence, it may only be partially dependent on a participant's language proficiency.^43^ Attention and executive functioning, as measured by the Trail Making Tests A and B, respectively, did not significantly predict emotion recognition, despite results from prior research which highlight an association between these cognitive domains and emotion recognition.^44,45^ One possible explanation is that, although these tasks involve executive control, they also rely on lower-level cognitive processes^46^ such as visual scanning and set-shifting.^47^ The absence of an association in our study may therefore reflect the multifactorial nature of this task rather than a lack of executive involvement in emotion recognition. Given that the performance of patients with MCI was comparable to that of the HCs across the cognitive domains in our study, we can rule out the influence of broad cognitive decline as a contributor to emotion recognition. Instead, emotion recognition difficulties in MCI may stem from more specific impairments in socio-emotional or perceptual processing rather than from generalized cognitive dysfunction.

### The association of emotional interference and emotion recognition

The study also revealed a significant association between emotional interference as measured by ESE reaction times and emotion recognition. Interestingly, this effect occurred independent of the general cognitive factors, which were not significant predictors in our models, hence highlighting the role of the automatic emotion-related processes that can affect perceptual tasks despite the absence of traditional top-down attentional models.^48^ The emotion-specific analysis suggested that disgust was less affected by the overall negative effect of ESE, which may be due to its evolutionary attachment to social norms and repulsion, which allows attention to the recognition of this emotion even in high-interference situations.^49^ However, at the group level, the results suggest that the association of interference with emotion recognition was similar in both groups, suggesting that while heightened interference is associated with lower emotion recognition, the impairments in patients with MCI could be explained by facial processing and emotional intelligence deficits rather than by interference alone.

### The association of emotional intelligence and emotion recognition

Finally, our study showed that emotional intelligence (EI) is significantly associated with emotion recognition in patients with MCI. Hence, emotional awareness enhances sensitivity to facial expressions and attention to emotional cues-especially since emotional expressions are known to capture attention more effectively than neutral ones.^50^ Previous research also shows that people who can connect their emotions to their thoughts are better at understanding the emotions of others.^51^ Moreover, individuals with high EI may be particularly attuned to subtle differences in emotional expressions, enabling them to more accurately perceive emotions by integrating multiple facial features.^52^ These findings suggest that deficits in EI may underlie difficulties in emotion recognition in MCI. Consequently, future studies and interventions should consider enhancing emotional awareness and emotion regulation in patients with MCI.

### Strengths and limitations

The study contributes to the existing literature on the importance of emotion recognition and the influence of an individual's cognitive status on this ability, particularly in patients with MCI. There are several strengths of this study, one of which is that it examines cognitive predictors and their contribution to emotion recognition while distinguishing between the performance of patients with MCI and healthy controls. Second, the study highlights the importance of holistic face processing in emotion recognition, thus consistent with previous studies that highlight the common neural pathways between face identity recognition and emotion recognition.^53^ These strengths would aid clinical efforts to develop targeted interventions that could assist patients with MCI in their interaction with others.

While the study provides valuable insights into emotion recognition deficits in patients with MCI, the limitations should be acknowledged. In our initial analysis plan, a priori power analysis suggested that 74 participants were needed, and due to recruitment constraints, specifically due to difficulties in recruiting patients with naMCI, we conducted a post hoc analysis and concluded data collection with 60 participants. While the sample size was sufficient for the preliminary analysis, future studies should include a more diverse sample (e.g., both aMCI and naMCI) to strengthen the generalizability of the findings. Second, caregiver burden data was collected using the Zarit Burden Interview (ZBI)^12^ to explore how the patient's emotion recognition deficits contributed to caregiver burden. However, this variable was not included in the final analysis because the data showed minimal variability as no participant scored more than a few points on the scale. Future studies should consider using a broader approach to capture caregiver burden in different contexts.

### Future directions

The results of this study provide several avenues for future research aimed at improving emotion recognition deficits in patients with MCI. Our use of composite face images that presented emotions on different halves of the face as a controlled approach allowed us to examine how patients with MCI process emotions from different facial regions. Future research could explore how videos (dynamic facial changes) would be able to reflect more real-world social interactions, where emotions unfold over time.^34^ Comparing results obtained from static and dynamic stimuli could provide more insight into the deficits concerning the emotion recognition abilities of those patients with MCI.

In terms of clinical intervention, one promising direction, for example, is the use of Ambulatory Assessment (AA), which could pave the way for assessing and incorporating emotion recognition training in the participant's natural environment. In addition, AA could be used to create mobile applications that prompt individuals to practice recognizing facial features, such as eyes and mouth, that are most salient for certain emotions.^54^ Such real-time interventions could improve emotion recognition, particularly for patients with MCI who have difficulty recognizing complex negative emotions, which require detailed processing of different facial regions.

### Conclusion

In summary, our study provides evidence that the emotion recognition deficits in MCI are particularly pronounced for negative emotions such as anger and are linked to impairments in face processing. Furthermore, our study demonstrated the association between EI and emotion recognition in patients with MCI, hence highlighting the need for further socio-emotional factor exploration in aging, especially for populations with cognitive decline. Future research should assess the impact of impaired emotion recognition on social interaction in an everyday context and aim to develop interventions, for example, using AA to support emotion recognition abilities in patients with MCI.

## Supplemental Material

sj-docx-1-alz-10.1177_13872877251414969 - Supplemental material for Emotion recognition in patients with mild cognitive impairment: The role of face processing and emotional intelligence Supplemental material, sj-docx-1-alz-10.1177_13872877251414969 for Emotion recognition in patients with mild cognitive impairment: The role of face processing and emotional intelligence by Rachana Mahadevan, Naomi Kristin Giesers, Thomas Liman, Karsten Witt, Andrea Hildebrandt and Mandy Roheger in Journal of Alzheimer's Disease
