# Supplementary material for: Emotion recognition in patients with mild cognitive impairment: The role of face processing and emotional intelligence
Source: J Alzheimers Dis. 2026 Feb 2;110(1):369–82. doi: 10.1177/13872877251414969 (PMC12960771; doi:10.1177/13872877251414969)
Supplement: sj-docx-1-alz-10.1177_13872877251414969 - Supplemental material for Emotion recognition in patients with mild cognitive impairment: The role of face processing and emotional intelligence [file sj-docx-1-alz-10.1177_13872877251414969.docx]

**Supplemental Material**

**Emotions recognition in patients with mild cognitive impairment: The role of face processing and emotional intelligence**

**Supplemental Table 1.** TREND Statement Checklist.

| **Paper Section/**  **Topic** | **Item No** | **Descriptor** |  |
| --- | --- | --- | --- |
|  |  |  | 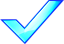 |
| Title and Abstract | 1 | 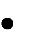 Information on how unit were allocated to interventions | ✓ |
|  |  | 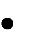 Structured abstract recommended | ✓ |
|  |  | 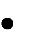 Information on target population or study sample | ✓ |
| Background | 2 | 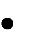 Scientific background and explanation of rationale | ✓ |
|  |  | 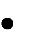 Theories used in designing behavioral interventions | ✓ |
| Participants | 3 | 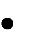 Eligibility criteria for participants, including criteria at different levels in recruitment/sampling plan (e.g., cities, clinics, subjects) | ✓ |
|  |  | Method of recruitment (e.g., referral, self-selection), including the sampling method if a systematic sampling plan was implemented 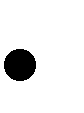 | ✓ |
|  |  | Recruitment setting 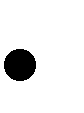 | ✓ |
|  |  | Settings and locations where the data were collected 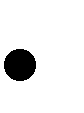 | ✓ |
| Interventions | 4 | 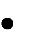 Details of the interventions intended for each study condition and how and when they were actually administered, specifically including: |  |
|  |  | - Content: what was given? |  |
|  |  | - Delivery method: how was the content given? |  |
|  |  | - Unit of delivery: how were the subjects grouped during delivery? |  |
|  |  | - Deliverer: who delivered the intervention? |  |
|  |  | - Setting: where was the intervention delivered? |  |
|  |  | - Exposure quantity and duration: how many sessions or episodes or events were intended to be delivered? How long were they intended to last? |  |
|  |  | - Time span: how long was it intended to take to deliver the   intervention to each unit? |  |
|  |  | - Activities to increase compliance or adherence (e.g., incentives) |  |
| Objectives | 5 | 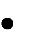 Specific objectives and hypotheses | ✓ |
| Outcomes | 6 | 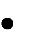 Clearly defined primary and secondary outcome measures | ✓ |
|  |  | 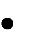 Methods used to collect data and any methods used to enhance the quality of measurements | ✓ |
|  |  | 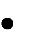 Information on validated instruments such as psychometric and biometric properties |  |
| Sample Size | 7 | How sample size was determined and, when applicable, explanation of any interim analyses and stopping rules 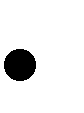 | ✓ |
| Assignment Method | 8 | Unit of assignment (the unit being assigned to study condition, e.g., individual, group, community) 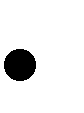 | ✓ |
|  |  | Method used to assign units to study conditions, including details of any restriction (e.g., blocking, stratification, minimization) 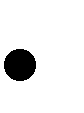 | ✓ |
|  |  | Inclusion of aspects employed to help minimize potential bias induced due to non-randomization (e.g., matching) 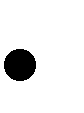 | ✓ |

| Blinding (masking) | 9 | 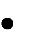 Whether or not participants, those administering the interventions, and those assessing the outcomes were blinded to study condition assignment; if so, statement regarding how the blinding was accomplished and how it was assessed. |  |
| --- | --- | --- | --- |
| Unit of Analysis | 10 | 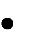 Description of the smallest unit that is being analyzed to assess intervention effects (e.g., individual, group, or community) |  |
|  |  | 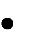 If the unit of analysis differs from the unit of assignment, the analytical method used to account for this (e.g., adjusting the standard error estimates by the design effect or using multilevel analysis) |  |
| Statistical Methods | 11 | 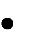 Statistical methods used to compare study groups for primary methods outcome(s), including complex methods of correlated data | ✓ |
|  |  | 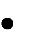 Statistical methods used for additional analyses, such as a subgroup analyses and adjusted analysis | ✓ |
|  |  | Methods for imputing missing data, if used 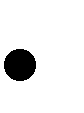 | ✓ |
|  |  | Statistical software or programs used 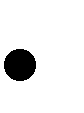 | ✓ |
| Participant flow | 12 | Flow of participants through each stage of the study: enrollment, assignment, allocation, and intervention exposure, follow-up, analysis (a diagram is strongly recommended) 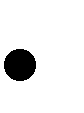 | ✓ |
|  |  | - Enrollment: the numbers of participants screened for eligibility, found to be eligible or not eligible, declined to be enrolled, and   enrolled in the study | ✓ |
|  |  | - Assignment: the numbers of participants assigned to a study   condition | ✓ |
|  |  | - Allocation and intervention exposure: the number of participants assigned to each study condition and the number of participants   who received each intervention | ✓ |
|  |  | - Follow-up: the number of participants who completed the follow- up or did not complete the follow-up (i.e., lost to follow-up), by   study condition |  |
|  |  | - Analysis: the number of participants included in or excluded from   the main analysis, by study condition | ✓ |
|  |  | 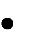 Description of protocol deviations from study as planned, along with reasons |  |
| Recruitment | 13 | Dates defining the periods of recruitment and follow-up 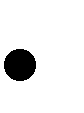 | ✓ |
| Baseline Data | 14 | Baseline demographic and clinical characteristics of participants in each study condition 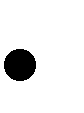 | ✓ |
|  |  | Baseline characteristics for each study condition relevant to specific disease prevention research 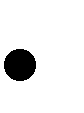 |  |
|  |  | Baseline comparisons of those lost to follow-up and those retained, overall and by study condition 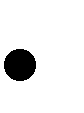 |  |
|  |  | Comparison between study population at baseline and target population of interest 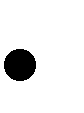 | ✓ |
| Baseline equivalence | 15 | - Data on study group equivalence at baseline and statistical methods used to control for baseline differences | ✓ |

| Numbers analyzed | 16 | 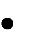 Number of participants (denominator) included in each analysis for each study condition, particularly when the denominators change for different  outcomes; statement of the results in absolute numbers when feasible |  |
| --- | --- | --- | --- |
|  |  | 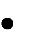 Indication of whether the analysis strategy was “intention to treat” or, if not, description of how non-compliers were treated in the analyses |  |
| Outcomes and estimation | 17 | 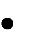 For each primary and secondary outcome, a summary of results for each estimation study condition, and the estimated effect size and a confidence interval to indicate the precision |  |
|  |  | 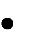 Inclusion of null and negative findings | ✓ |
|  |  | Inclusion of results from testing pre-specified causal pathways through which the intervention was intended to operate, if any 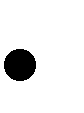 |  |
| Ancillary  analyses | 18 | 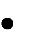 Summary of other analyses performed, including subgroup or restricted analyses, indicating which are pre-specified or exploratory |  |
| Adverse events | 19 | 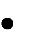 Summary of all important adverse events or unintended effects in each study condition (including summary measures, effect size estimates, and  confidence intervals) | ✓ |
| Interpretation | 20 | 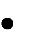 Interpretation of the results, taking into account study hypotheses, sources of potential bias, imprecision of measures, multiplicative analyses,  and other limitations or weaknesses of the study | ✓ |
|  |  | Discussion of results taking into account the mechanism by which the intervention was intended to work (causal pathways) or alternative mechanisms or explanations 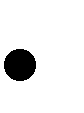 | ✓ |
|  |  | Discussion of the success of and barriers to implementing the intervention, fidelity of implementation 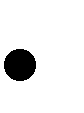 | ✓ |
|  |  | Discussion of research, programmatic, or policy implications 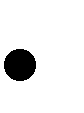 | ✓ |
| Generalizability | 21 | 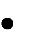 Generalizability (external validity) of the trial findings, taking into account the study population, the characteristics of the intervention, length of follow-up, incentives, compliance rates, specific sites/settings involved in  the study, and other contextual issues | ✓ |
| Overall  Evidence | 22 | 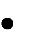 General interpretation of the results in the context of current evidence and current theory | ✓ |

**Supplemental Table 2**. The following table describes the sub-tests belonging to various domains of CERAD – NAB.

| Domains | Subtests | Test Description |
| --- | --- | --- |
| Attention | Trail Making Test A (TMT A) | Participants were instructed to connect a set of 25 dots as soon as possible, and the time for completing was then recorded by us. |
| Executive functioning | Trail Making Test B (TMT B) | Participants had to connect circles in an ascending pattern, however, unlike TMT A, where the participants had to alternate between numbers and letters, for example, 1-A-2-B-3-C, etc., and we recorded the time they took to complete this task. |
| Memory | Word list learning test | This test is used to assess the participant’s immediate memory skills where we showed the participant a list of 10 words in three different trials and they had to read the words out loud with a gap of 2 seconds between each word. The participant had to recall as many words as possible after each trial within 90 seconds. The number of words they could recall was then noted down by us. |
|  | Word list recall test | This test was administered after a few minutes of completing the word list learning test to assess the delayed memory of the participant in this test, they had to recall as many previously learned words as possible within 90 seconds. The number of words recalled is then once again noted down by us. |
|  | Word recognition test | This test is similar to the word recall test and was intended to assess the delayed memory skills of the participants, here they were presented with a list of words and were instructed to point out if these words were a part of the previously learned list or not. This list consisted of twenty  words out of which 10 words were a part of the initial list and the other 10 were not. Their performance in this task was recorded by us with the help of a given checklist. |
| Language | Verbal fluency test | In this task, the participant had to name as many words as possible that were possible from a given category (‘animals’) within 60 seconds. |
|  | Boston naming test | In this test, the participant was shown pictures of objects and they had to recognize the objects. As and when the objects were recognized their responses were recorded in the given checklist. |
|  | Phonematic Fluency | In this test, participants had to name as many words as possible for a given alphabet (‘S’) within 60 seconds and their responses were written down. |
| Visuospatial construction | Figure construction test | In this test, the participant had to copy a figure provided to them (circle, rhombus, intersecting rectangles, and a cube). This was then assessed for the shape and figure lines based on a given criteria. |
| Visuospatial memory | Figure recall test | In this test, the participants had to recall as many figures as they could from the figure construction test and draw them on a blank sheet of paper. |

**Supplemental Table 3**. The following table describes the interpretation of the scores of the Trait Emotional Intelligence Questionnaire (TEIQUE - SF).

| **Domain** | **High scores** | **Low scores** |
| --- | --- | --- |
| Well-Being | Higher scores in well-being can indicate that the participants tend to have a high sense of well-being and that it can be drawn from past achievements to be attributed to future expectations. | Participants who had low scores tend to have low self-regard which ultimately leads to disappointment about their lives. |
| Self-control | High scores in this category indicate that the participants can control their impulses, urges, and desires to a great extent while also being good at regulating factors such as external pressure and stress that accompany these urges. | Participants who scored less in this category tend to have highly impulsive behaviors and are unable to manage the stress that accompanies it. |
| Emotionality | High scores in emotionality indicated that according to participants they have no difficulties in perceiving the emotions of others while also being able to express their emotions, hence being able to use these skills to develop and maintain relationships with their family and friends. | Participants who scored less generally find it difficult to recognize the emotions of others while also not being able to express the emotions they are experiencing hence leading to tension in their relationships. |
| Sociability | High scores in sociability indicate that the participants are relatively good during social interactions due to their good listening skills and efficient communication skills. | Participants with low scores do not have communication skills efficient enough to be good negotiators hence resulting in them being more reserved and shyer in approaching other people. |

**Supplemental Table 4.** Frequency tables for the Groups (HC versus MCI).

| **Group** | **Count** | **Percentage (%)** |
| --- | --- | --- |
| 0 | 30 | 50 |
| 1 | 30 | 50 |

**Supplemental Table 5.** Frequency tables for Gender (Female versus Male).

| **Gender** | **Count** | **Percentage (%)** |
| --- | --- | --- |
| Female | 33 | 55 |
| Male | 27 | 45 |

**Supplemental Table 6.** Association of language tests on ECT performance

| **Parameter** | **β** | **CI** | ***p*** | **FDR *p*** |
| --- | --- | --- | --- | --- |
| (Intercept) | 0.17 | [-0.21, 0.56] | **<0.001** | **<0.001** |
| Verbal fluency | -0.28 | [-0.72, 0.17] | 0.217 | 0.395 |
| Boston naming test | -0.01 | [-0.42, 0.40] | 0.955 | 0.955 |
| Phonematic fluency | 0.32 | [-0.14, 0.78] | 0.165 | 0.395 |
| Group | -0.35 | [-0.93, 0.23] | 0.271 | 0.395 |
| Verbal fluency x Group | 0.27 | [-0.31, 0.85] | 0.361 | 0.464 |
| Boston naming test x Group | 0.32 | [-0.22, 0.86] | 0.240 | 0.395 |
| Phonematic fluency x Group | -0.14 | [-0.71, 0.43] | 0.616 | 0.693 |

Residual standard error: 13.94 on 51 degrees of freedom, Multiple R-squared: 0.1733, Adjusted R-squared: 0.0436

F-statistic: 1.336 on 8 and 51 DF, p-value: 0.2474.

β displays the standardized β values, FDR is the false discovery rate, Confidence Interval (CI) =95%, and statistically significant results are shown in bold (p<0.001 and p<0.05). The Verbal Fluency, Boston Naming Test, and Phonemic Fluency subtests assess the language domain within the neuropsychological test battery. The group variable compares the emotion recognition abilities of the patients with mild cognitive impairment (MCI) and healthy controls (HC). The interaction between each language test and the groups was used to assess whether language abilities contributed to better emotion recognition.

**Supplemental Table 7.** Association of memory tests on ECT performance.

| **Parameter** | **β** | **CI** | ***p*** | **FDR *p*** |
| --- | --- | --- | --- | --- |
| (Intercept) | -0.01 | [-0.54, 0.52] | **<0.001** | **<0.001** |
| Immediate memory | 0.01 | [-0.44, 0.46] | 0.960 | 0.960 |
| Group | -0.02 | [-0.78, 0.73] | 0.439 | 0.629 |
| Delayed memory | -0.18 | [-0.64, 0.27] | 0.423 | 0.629 |
| Word recognition | -0.14 | [-0.59, 0.31] | 0.537 | 0.657 |
| Visuospatial recall | 0.66 | [0.05, 1.28] | **<0.05** | 0.197 |
| Age | -0.16 | [-0.46, 0.13] | 0.269 | 0.629 |
| Immediate memory x Group | 0.04 | [-0.69, 0.78] | 0.906 | 0.960 |
| Delayed memory x Group | 0.23 | [-0.38, 0.83] | 0.457 | 0.629 |
| Word recognition x Group | 0.26 | [-0.34, 0.85] | 0.390 | 0.629 |
| Visuospatial recall x Group | -0.49 | [-1.18, 0.20] | 0.160 | 0.588 |

Residual standard error: 14.06 on 49 degrees of freedom, Multiple R-squared: 0.192, Adjusted R-squared: 0.027

F-statistic: 1.166 on 10 and 49 DF, p-value: 0.336.

β displays the standardized β values, FDR is the false discovery rate, Confidence Interval (CI) =95%, and statistically significant results are shown in bold (p<0.001 and p<0.05). The Immediate memory, Delayed memory, Word recognition, and Visuospatial recall subtests assess the memory domain within the neuropsychological test battery. The group variable compares the emotion recognition abilities of the patients with mild cognitive impairment (MCI) and healthy controls (HC). The interaction between each memory test and the groups was used to assess whether memory abilities contributed to better emotion recognition.

**Supplemental Table 8.** Association of the visuospatial construction test on the ECT performance.

| **Parameter** | **β** | **CI** | ***p*** | **FDR *p*** |
| --- | --- | --- | --- | --- |
| (Intercept) | 0.09 | [-0.31, 0.49] | **<0.001** | **<0.001** |
| Visuospatial construction | 0.32 | [-0.19, 0.83] | 0.209 | 0.522 |
| Group | -0.22 | [-0.80, 0.36] | 0.451 | 0.563 |
| Age | -0.12 | [-0.4, 0.16] | 0.405 | 0.563 |
| Visuospatial construction x Group | -0.13 | [-0.72, 0.47] | 0.668 | 0.668 |

Residual standard error: 13.86 on 55 degrees of freedom, Multiple R-squared: 0.1189, Adjusted R-squared: 0.05483

F-statistic: 1.856 on 4 and 55 DF, p-value: 0.1314.

β displays the standardized β values, FDR is the false discovery rate, Confidence Interval (CI) =95%, and statistically significant results are shown in bold (p<0.001 and p<0.05). Visuospatial construction tests assess the visuospatial construction abilities within the neuropsychological test battery. The group variable compares the emotion recognition abilities of the patients with mild cognitive impairment (MCI) and healthy controls (HC). The interaction between the visuospatial construction and the groups was used to assess whether this ability contributed to better emotion recognition.

**Supplemental Table 9.** Association of attention tests on ECT performance.

| **Parameter** | **β** | **CI** | ***p*** | **FDR *p*** |
| --- | --- | --- | --- | --- |
| (Intercept) | 0.19 | [-0.19, 0.58] | **<0.001** | **<0.001** |
| TMT A | -0.16 | [-0.53, 0.21] | 0.388 | 0.450 |
| Group | -0.36 | [-0.94, 0.21] | 0.101 | 0.251 |
| Age | -0.12 | [-0.44, 0.20] | 0.450 | 0.450 |
| TMTA x Group | 0.30 | [-0.22, 0.82] | 0.260 | 0.433 |

Residual standard error: 14.09 on 55 degrees of freedom, Multiple R-squared: 0.08968, Adjusted R-squared: 0.02348

F-statistic: 1.355 on 4 and 55 DF, p-value: 0.2616.

β displays the standardized β values, FDR is the false discovery rate, Confidence Interval (CI) =95%, and statistically significant results are shown in bold (p<0.001 and p<0.05). TMT A refers to the Trail Making Test A test assessing attention abilities within the neuropsychological test battery. The group variable compares the emotion recognition abilities of the patients with mild cognitive impairment (MCI) and healthy controls (HC). The interaction between attention and the groups was used to assess whether this ability contributed to better emotion recognition.

**Supplemental Table 10.** Association of the executive functioning test on the ECT performance

| **Parameter** | **β** | **CI** | ***p*** | **FDR *p*** |
| --- | --- | --- | --- | --- |
| (Intercept) | 0.16 | [-0.22, 0.54] | **<0.001** | **<0.001** |
| TMT B | -0.15 | [-0.49, 0.18] | 0.362 | 0.362 |
| Group | -0.35 | [-0.92, 0.22] | 0.103 | 0.257 |
| Age | -0.13 | [-0.42, 0.15] | 0.357 | 0.362 |
| TMTB x Group | 0.31 | [-0.22, 0.84] | 0.249 | 0.362 |

Residual standard error: 14.08 on 55 degrees of freedom, Multiple R-squared: 0.09091, Adjusted R-squared: 0.02479

F-statistic: 1.37 on 4 and 55 DF, p-value: 0.2545

β displays the standardized β values, FDR is the false discovery rate, Confidence Interval (CI) =95%, and statistically significant results are shown in bold (p<0.001 and p<0.05). TMT B refers to the Trail Making Test B test, which assesses executive functioning abilities within the neuropsychological test battery. The group variable compares the emotion recognition abilities of the patients with mild cognitive impairment (MCI) and healthy controls (HC). The interaction between executive functioning and the groups was used to assess whether this ability contributed to better emotion recognition.

**Supplemental Table 11.** Gender and stimuli interaction on ECT performance.

| **Parameter** | **β** | **CI** | **p** | **FDR p** |
| --- | --- | --- | --- | --- |
| (Intercept) | 0.00 | [-0.04, 0.03] | 0.660 | 0.754 |
| Gender | 0.00 | [-0.04, 0.04] | 0.557 | 0.754 |
| Group | 0.01 | [-0.03, 0.06] | 0.589 | 0.754 |
| Male stimuli | 0.57 | [0.53, 0.62] | **<0.001** | **<0.001** |
| Female stimuli | 0.49 | [0.45, 0.53] | **<0.001** | **<0.001** |
| Age | 0.00 | [-0.02, 0.02] | 0.969 | 0.969 |
| Gender x Male stimuli | -0.04 | [-0.10, 0.02] | 0.168 | 0.335 |
| Gender x Female stimuli | 0.05 | [-0.01, 0.11] | 0.088 | 0.235 |

Residual standard error: 1.082 on 52 degrees of freedom, Multiple R-squared: 0.9949, Adjusted R-squared: 0.9942

F-statistic: 1455 on 7 and 52 DF, p-value: < 2.2e-16.

β displays the standardized β values, FDR is the false discovery rate, Confidence Interval (CI) =95%, and statistically significant results are shown in bold (p<0.001 and p<0.05). The group variable compares the emotion recognition abilities of the patients with mild cognitive impairment (MCI) and healthy controls (HC). The gender variable compares the emotion recognition abilities of different genders who participated in the study. The interaction between participant gender and stimulus gender was used to assess whether different participant genders were influenced by different stimulus genders.

**Supplemental Table 12.** Association of demographic factors on ECT performance.

| **Parameter** | **β** | **CI** | ***p*** | **FDR *p*** |
| --- | --- | --- | --- | --- |
| (Intercept) | 0.34 | [-0.22, 0.90] | 0.214 | 0.952 |
| Age | 0.02 | [-0.45, 0.50] | 0.919 | 0.952 |
| Group | -0.28 | [-1.06, 0.49] | 0.905 | 0.952 |
| Education | 0.20 | [-0.29, 0.69] | 0.422 | 0.952 |
| Gender | -0.35 | [-1.18, 0.49] | 0.406 | 0.952 |
| Age x Group | -0.08 | [-0.72, 0.56] | 0.813 | 0.952 |
| Education x Group | -0.02 | [-0.62, 0.58] | 0.952 | 0.952 |
| Gender x Group | -0.10 | [-1.25, 1.04] | 0.858 | 0.952 |

Residual standard error: 14.26 on 52 degrees of freedom, Multiple R-squared: 0.1184, Adjusted R-squared: -0.0002938

F-statistic: 0.9975 on 7 and 52 DF, p-value: 0.4437.

β displays the standardized β values, FDR is the false discovery rate, Confidence Interval (CI) =95%, and statistically significant results are shown in bold (p<0.001 and p<0.05). The group variable compares the emotion recognition abilities of the patients with mild cognitive impairment (MCI) and healthy controls (HC). The gender variable compares the emotion recognition abilities of different genders who participated in the study. Education compares the emotion recognition abilities of those belonging to various levels of education. The interaction between age, gender, education, and group was the assessment of the contribution of these factors in recognizing emotions across groups.

**
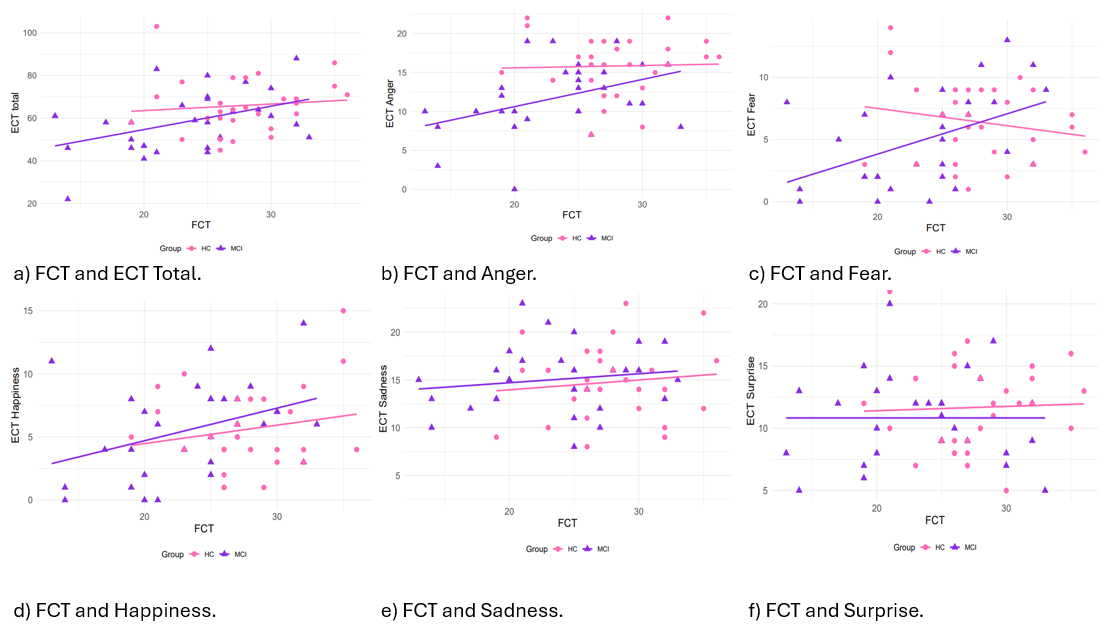
**

**
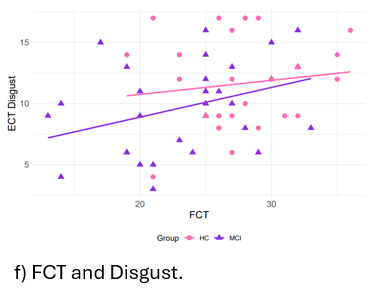
**

**Supplemental Figure 1.** Scatterplots of ECT scores across FCT scores, comparing group performance (MCI versus HC) for each emotion. Key: = Patients with MCI, = Healthy Controls.


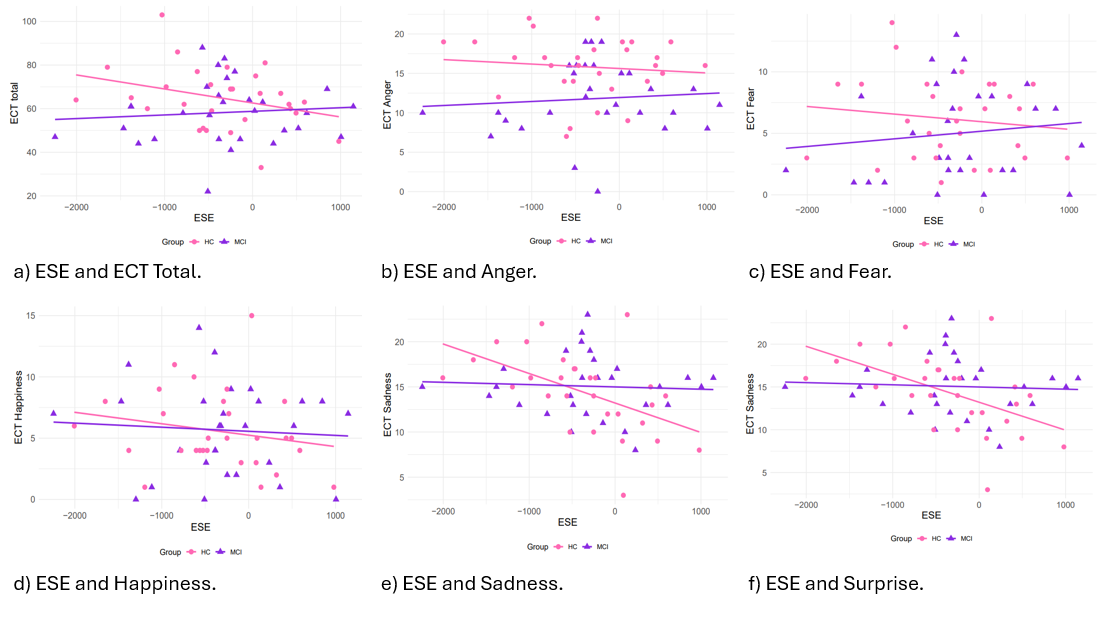


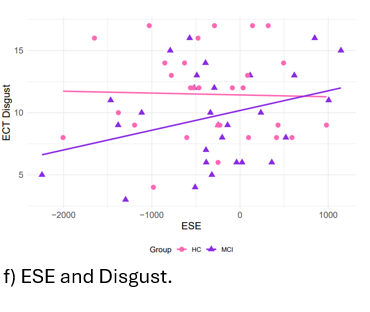


**Supplemental Figure 2.** Scatterplots of ECT scores across ESE reaction times, comparing group performance (MCI versus HC) for each emotion. Key: = Patients with MCI, = Healthy Controls.


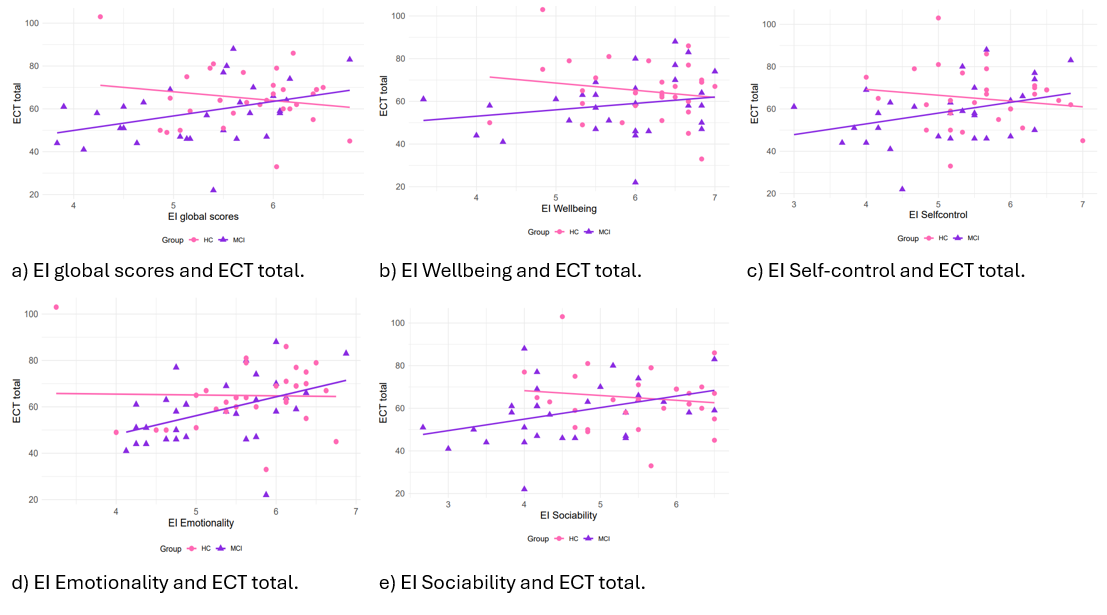


**Supplemental Figure 3.** Scatterplots of total ECT scores across scores on the EI global scores and its subscales, comparing group performance (MCI versus HC). Key: = Patients with MCI, = Healthy Controls.

**
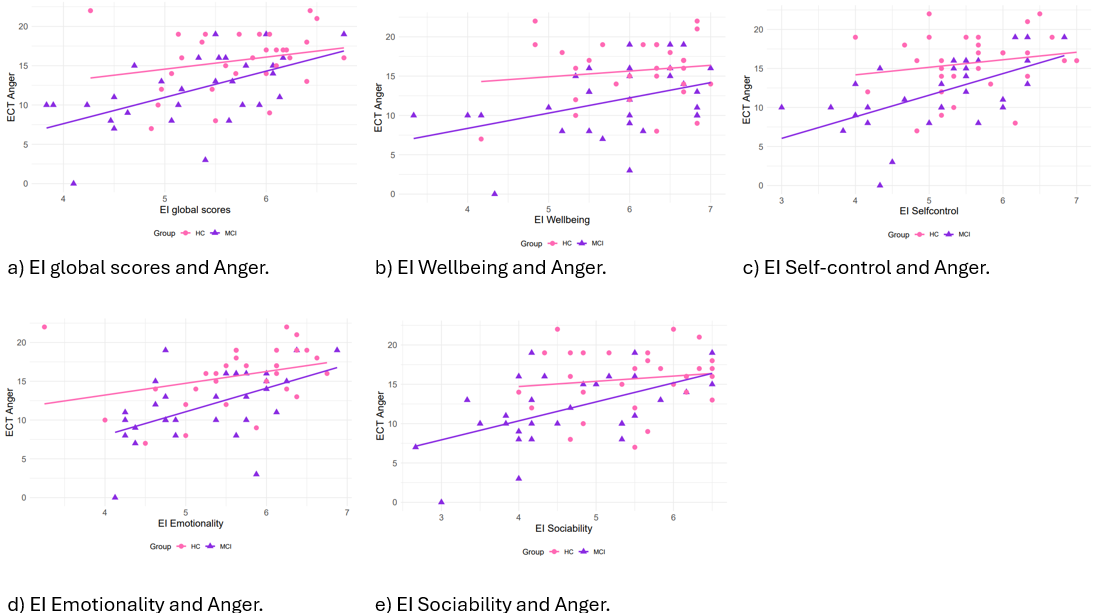
**

**Supplemental Figure 4.** Scatterplots of Anger ECT scores across scores on the EI and its subscales, comparing group performance (MCI versus HC). Key: = Patients with MCI, = Healthy Controls.


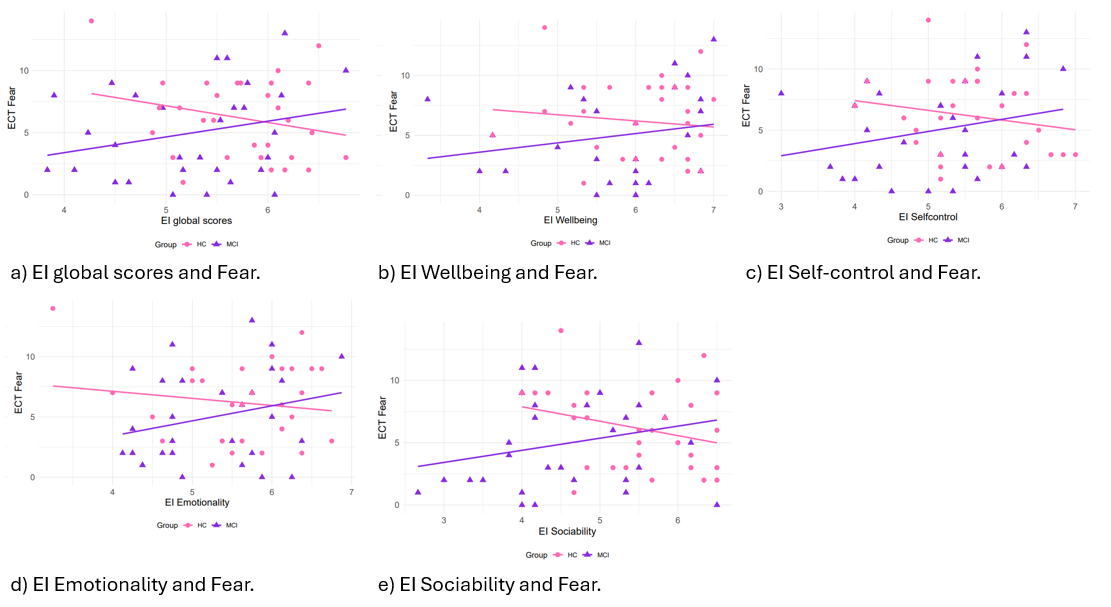


**Supplemental Figure 5.** Scatterplots of Fear ECT scores across scores on the EI and its subscales, comparing group performance (MCI versus HC). Key: = Patients with MCI, = Healthy Controls.


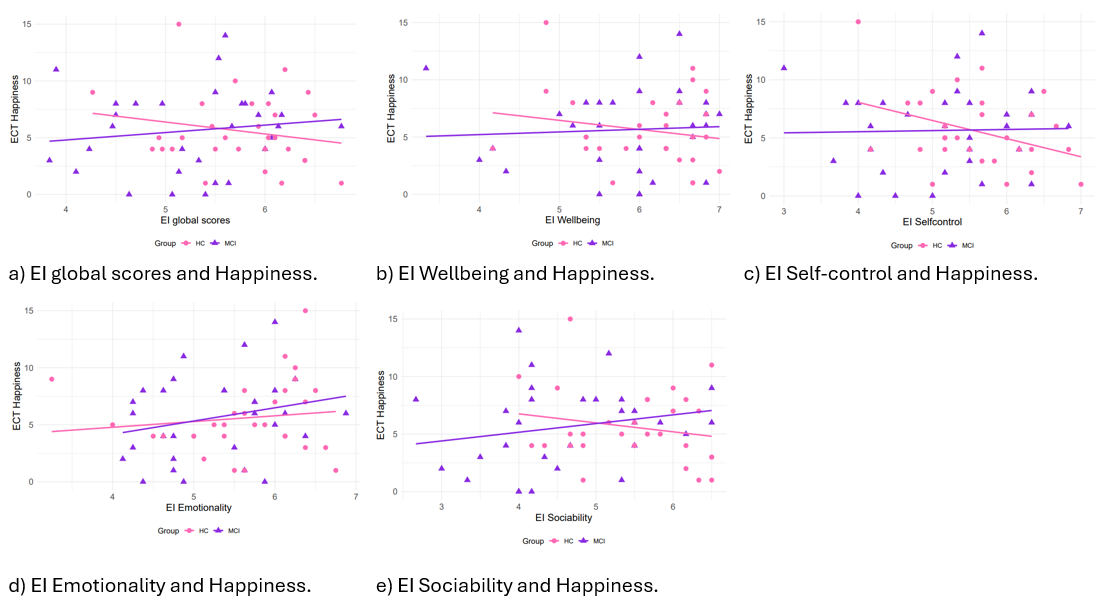


**Supplemental Figure 6.** Scatterplots of Happiness ECT scores across scores on the EI and its subscales, comparing group performance (MCI versus HC). Key: = Patients with MCI, = Healthy Controls.


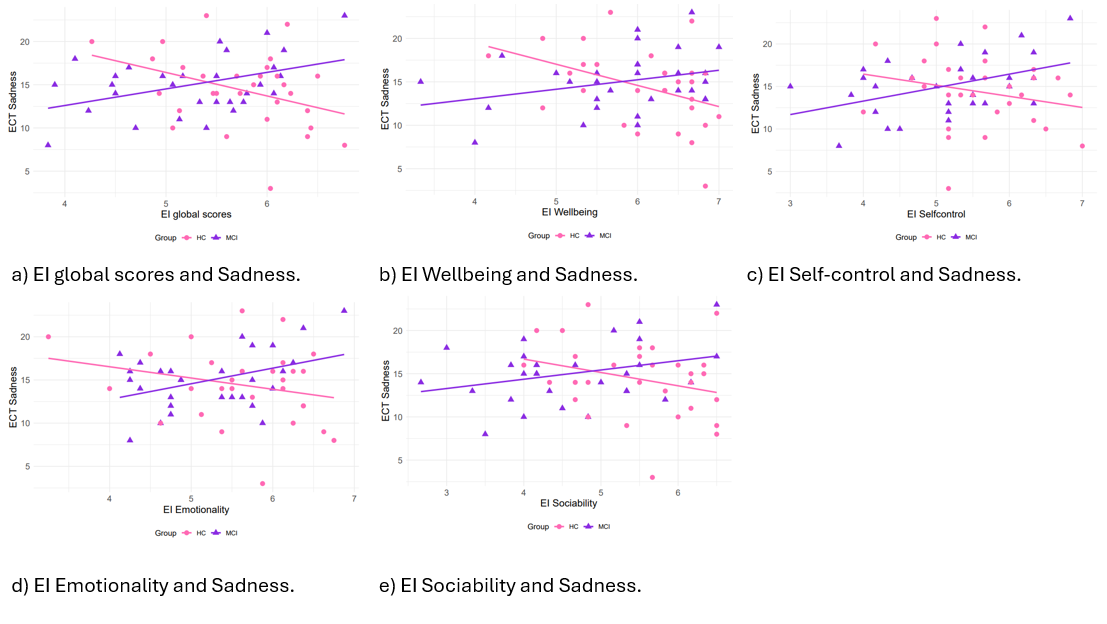


**Supplemental Figure 7.** Scatterplots of Sadness ECT scores across scores on the EI and its subscales, comparing group performance (MCI versus HC). Key: = Patients with MCI, = Healthy Controls.


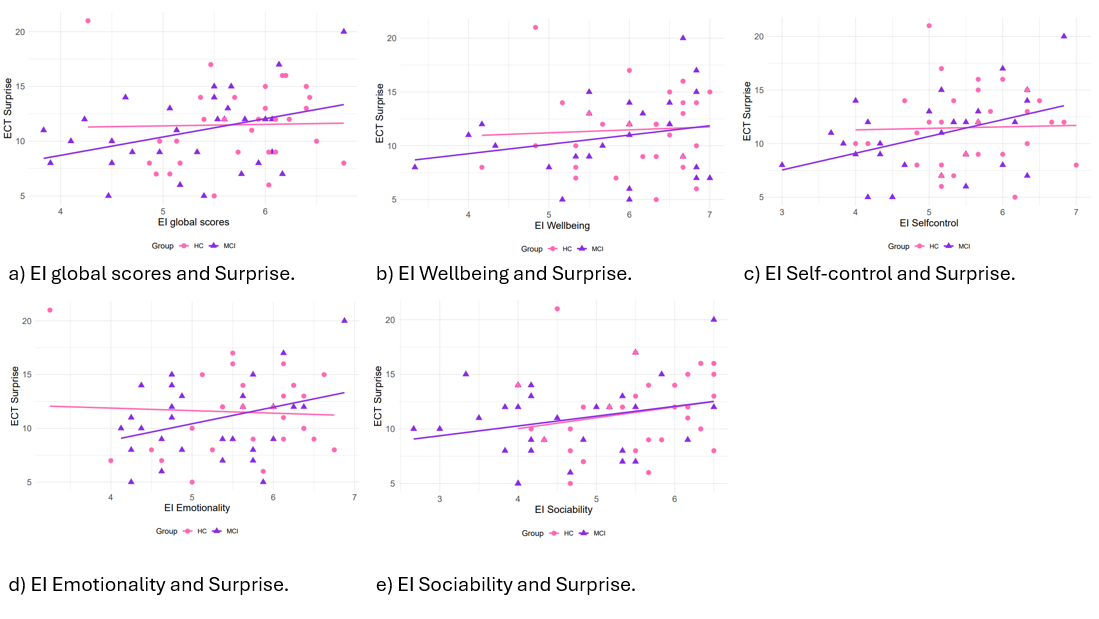


**Supplemental Figure 8.** Scatterplots of Surprise ECT scores across scores on the EI and its subscales, comparing group performance (MCI versus HC). Key: = Patients with MCI, = Healthy Controls.


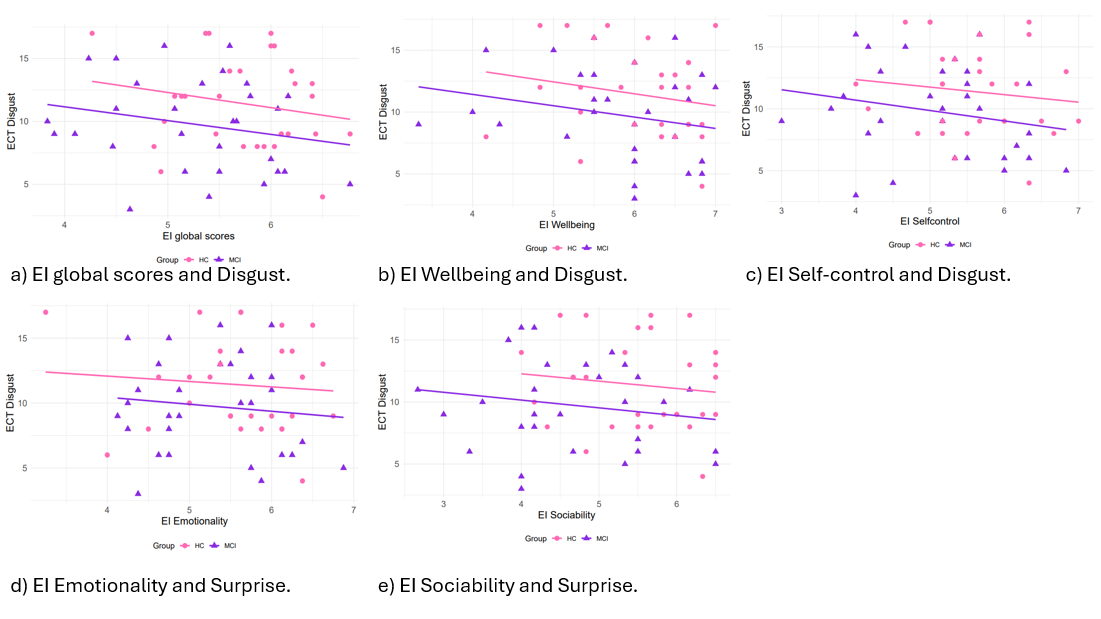


**Supplemental Figure 9.** Scatterplots of Disgust ECT scores across scores on the EI and its subscales, comparing group performance (MCI versus HC). Key: = Patients with MCI, = Healthy Controls.

**Additional text**

*Neuropsychological assessment*

As a part of the screening, we administered the Instrumental Activities of Daily Living (IADL). The goal of this tool is to assess the participant’s ability to independently perform activities such as the use of the telephone, shopping for groceries, cooking, chores, laundry, and the use of transportation. If the participant could perform an activity on their own, they were assigned one point, and if they could not perform an activity on their own and needed assistance, they were assigned 0 points. A maximum score of 8 points can be obtained, and a minimum score of 0 can be obtained. If a participant scores below 6, then it can be concluded that he or she had difficulties functioning independently while performing day-to-day activities.

To detect anxiety or depression, we administered the Hospital Anxiety and Depression Scale (HADS). The tool has 21 questions out of which 7 items address anxiety and 7 items address depression. The tool was directly filled in by the participant, where they had to read the given statement and rate their responses between ‘Often/most of the time’ to ‘not at all/very seldom’ based on how they had been feeling in the past week. For each question, a maximum of 3 points or a minimum of 0 points can be allotted. For the tool as a whole, a maximum score of 21 and a minimum score of 0 can be obtained for each category.

To differentiate between the healthy controls and patients with MCI, we administered the Montreal Cognitive Assessment (MoCA). The MoCA is a rapid screening tool that is shown to have a sensitivity of 90% and a specificity of 87% in detecting MCI. In this tool, the participants were administered tests of various cognitive domains such as attention, memory, language, visuospatial construction, and executive functioning. The tool comes with established guidelines that highlight the instructions and scoring criteria. A total score of 30 can be obtained. Participants who scored between the range of 18 to 25 were classified as patients with MCI, and participants who scored above 26 and above were classified as healthy controls. Since the Peterson criteria does not specify a neuropsychological battery to test MCI, we applied the Consortium to Establish a Registry for Alzheimer’s disease Neuropsychology battery (CERAD-NAB) which is the most commonly used test in the German-speaking part of Europe and has been adjusted according to the age, gender and education for an elderly German population.
